# Imaging technique for the complete edentulous patient treated conventionally or with mini implant overdenture

**Published:** 2013-03-25

**Authors:** M Meleşcanu Imre, E Preoteasa, AM Țâncu, CT Preoteasa

**Affiliations:** * Department of Prosthodontics, Faculty of Dental Medicine, “Carol Davila" University of Medicine and Pharmacy; ** Department of Oral Diagnosis, Ergonomics, Scientific Research Methodology, Faculty of Dental Medicine, “Carol Davila" University of Medicine and Pharmacy

**Keywords:** edentulous, overdenture, teleradiography, mini dental implants

## Abstract

**Rationale.** The imaging methods are more and more used in the clinical process of modern dentistry. Once the implant based treatment alternatives are nowadays seen as being the standard of care in edentulous patients, these techniques must be integrated in the complete denture treatment.

** Aim.** The study presents some evaluation techniques for the edentulous patient treated by conventional dentures or mini dental implants (mini SKY Bredent) overdentures, using the profile teleradiography. These offer data useful for an optimal positioning of the artificial teeth and the mini dental implants, favoring to obtain an esthetic and functional treatment outcome. We proposed also a method to conceive a simple surgical guide that allows the prosthetically driven implants placement.

** Material and method.** Clinical case reports were made, highlighting the importance of cephalometric evaluation on lateral teleradiographs in complete edentulous patients. A clinical case that gradually reports the surgical guide preparation (Bredent silicon radio opaque), in order to place the mini dental implants in the best prosthetic and anatomic conditions, was presented.

** Conclusions.** The profile teleradiograph is a useful tool for the practitioner. It allows establishing the optimal site for implant placement, in a good relation with the overdenture. The conventional denture can be easily and relatively costless transformed in a surgical guide used during implant placement.

## Introduction

Nowadays, the imaging techniques are frequently used in dental removable prosthodontics, especially for implant-based treatment alternatives. They represent mandatory paraclinical exams in implantology, giving the information needed in order to establish the optimal treatment plan (i.e., number and place for the dental implants, their length and diameter). 

 The radiographic techniques usually used in prosthodontics on implants are the following: orthopantomography, teleradiography, computerized tomography, CBCT. Orthopantomography gives the doctor general information regarding the available bone volume and the neighborhood relationships with the anatomical structures –i.e. the maxillary sinus, the mandibular nerve path and nasal fosses. Another imaging investigation that provides more data and is used frequently in implantology in order to obtain an accurate evaluation is the computer tomography technique. This method provides also qualitative data regarding the bone, respectively the bone density. 

 Besides these imaging techniques frequently used in implantology, sometimes frontal and lateral teleradiography are used for cephalometric analysis. The latter are mainly used in orthodontics, but have applicability also in full edentulism patient evaluation [**1**]. Lateral teleradiography allows the complex visualization of the bone outlines in relation to the soft tissues, like the lips, important elements in positioning the teeth from the esthetic point of view, with consequences on complete denture`s balance.

### Objective

The study aims to present some evaluation techniques for the edentulous patient, treated by conventional dentures or mini dental implants overdentures, using the lateral teleradiography. These offer data useful for an optimal positioning of the artificial teeth and the mini dental implants, favoring to obtain an esthetic and functional treatment outcome. We proposed also a method to conceive a simple surgical guide which allows the prosthetically driven implants placement in accordance with the criteria mentioned before.

## Matherial and method

Cranial teleradiography is standardized and reproducible radiography of the cranium used especially in orthodontics to analyze the relationship between teeth and maxilla and between maxilla and the bones of the facial massive. With it can be done a better prognosis, more exact and dynamic, offering details about the most adequate treatment, and it’s efficacy in time.

 In 1960, Skotnycky lays out the teleradiographie’s value from the prosthodontic point of view, showing that an anatomical evaluation of the visceral cranium (skull) can be done, knowing the normal profile, which may represent an important guide for the dento-facial reconstructions that will be done. The skull teleradiography can be done in 3 positions: latero-lateral, postero-anterior and axial [**2**].

 Sagital and frontal teleradiographies can be done before or during the treatment, with occlusal rims, mock-ups, dentures. Before, in relation to the investigated areas, the base or denture’s teeth, have to be radio opaque. The periodical over positioning of the negatives allows the evaluation of the prosthetic treatment its failure and the causes of them can be discovered.

**Fig. 1:**
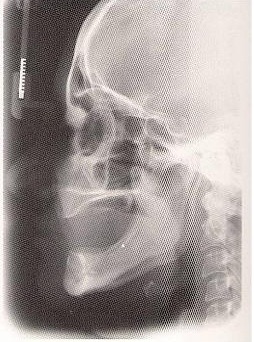
Profile teleradiography of a complete edentulous patient

 The profile teleradiography allows the comparative study of the soft parts and osseous infrastructure (Fig.1) [**3**]. 

 In order to obtain on the film the equal dimensions with the real one is necessary that the distance between the radiogenic tube and the object (the medio-sagital plane of the head in the profile teleradiography) is 3-5 cm, and the distance between the object and the radiologic film is 14 mm (Castano şi Chateau, 1960 citied by Firu). 

 In the wake of gnato- and cephalometric analysis, we can conclude about the intern and extern morphology, the teleradiography being helpful in establishing a more complete diagnosis [**1**]. 

**Fig. 2:**
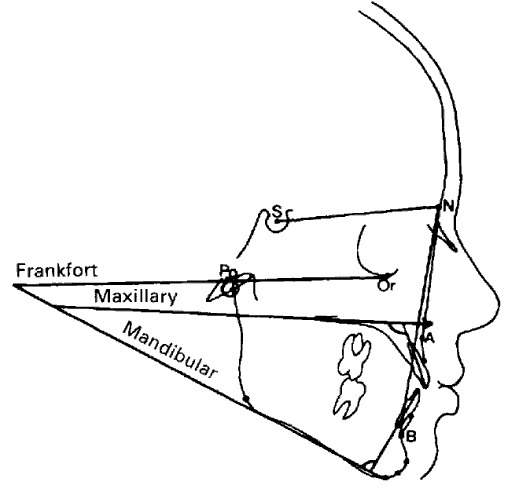
Plans and cefalometric points

 Current methods of teleragiography interpretation in prosthodontic treatment

 The cranio-facial structures and the relationships between them are evaluated trough the measurement of the distances, angles and proportions between different cephalometric layouts (**Fig. 2**). The anthropometric points can be osseous, dental, dermic, obtained through the attentive observation of the anatomical structures, or built through the intersection of lines and layouts. There are many interpretation methods of the profile teleradiography: Sassouni, Ricketts, Steiner, depending of the area of interest.

 As we mentioned before, the major problem in the use of profile teleradiography in full edentulism is the radiopacisation of the denture’s elements, the acrylic base and artificial teeth. In order to visualize them, different methods can be used, that will be illustrated. The first method consists in laying on the artificial teeth of a thin film of aluminium that allows the visualization of the artificial arcades. This type of teleradiography gives to the doctor information regarding the occlusion relationships in the front area reported to the osseous markers and the relationships with the lips and soft parts, aspect that will described in a suite of clinical cases.

 Case 1 – The imaging evaluation using the profile teleradiography of the correctness of the conventional denture at the full edentulous patient. 

 With the use of the teleradiograpgy, corroborated with the facial clinical exam, the former dentures were evaluated, from the normal and profile view. The profile teleradiography was done following the next protocol: the patient has his head in the cefalostat, with the dentures in the oral cavity, within teeth in contact (occlusion), with the relaxed muscles, not to modify the facial soft parts. The radiologic highlight of the artificial dental arcades and the occlusal relationships between the dentures was possible applying a thin lead layer of 2 mm on the outline of the teeth.

 Profile teleradiography with the old and new dentures

**Fig. 3:**
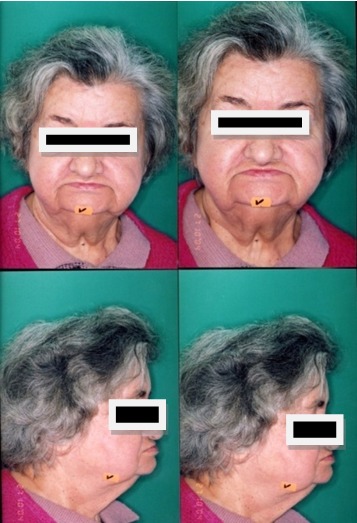
Facial oral exam – front and profile- old dentures

 Patient M.M,, 85 years old, full edentulous with conventional total dentures, with incorrect old dentures with unsatisfactory esthetic aspect [**4**]. Thus, one can observe the plugged aspect of the lips, the prominent chin (menton) and the lack of proportion between the face’s parts (**Fig. 3**).

**Fig. 4:**
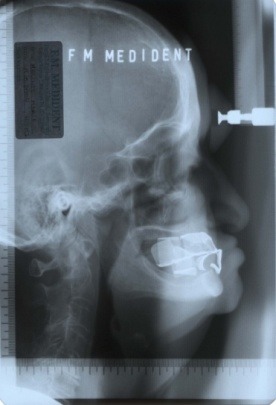
Profile teleradiography with the old dentures

 The observations of the clinical image of the face with the old dentures and the incorrect occlusal relationships in the front are confirmed by the cephalometric exam and analysis, aspect that has to be redressed (**Fig. 4**).

**Fig. 5:**
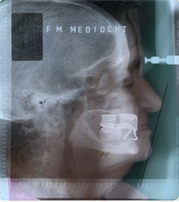
The overpositioning of the forography over the profile teleradiograph

**Fig. 6:**
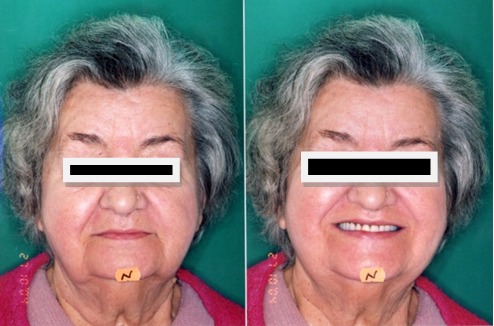
Oral exam with the new dentures

More than this, the modern imaging techniques allow the over position of the photographic image over the profile teleradiography in order to verify the integrity of the dental arches with the soft face’s parts (**Fig. 5**). Orofacial exam with the new dentures from the front and profile (**Fig. 6**).

**Fig. 7:**
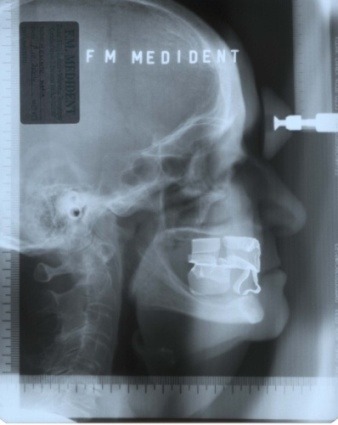
Profile teleradiography with the new dentures

 After the new dentures’ achievement, we can observe clinical the harmonious renewal of the face, according to the age of the patient, the normal lips step, the correct proportions between the face’s parts and an agreeable visibility of the teeth in the smile dynamic. Profile teleradiography with the new dentures in the mouth (**Fig. 7**).

 Case 2. The imaging evaluation of the full edentulous for overdentures on implants and mini implants

 Imaging evaluation of the complete edentulous for the overdentures on implants and mini implants treatment

 Another case, intended for demonstrating another applicability of the imaging techniques, respective the contribution of the profile teleradiography in implantology and full overdentures. According to the implanto-prosthodontic standards, the implant’s placement is prosthodonticly guided. More than that, in the overdentures cases is necessary to evaluate the space needed for the implants, to respond at the balance criteria of the conventional full prosthodontic treatment (function and esthetics), but also the space needed for the special anchorage elements of the overdenture.

 Patient A.L., 42 years old, came to our Prosthodontic Dental Clinic, Department of Full Edentulous Clinic of UMF Carol Davila to be treated suffering of a subtotal maxillary edentulism and a mandibular full edentulism, treated with a unsatisfactory denture. The patient was dissatisfied because of the unsatisfactory chewing and physionomics deficiencies with the conventional dentures. She was treated with a maxillary overdenture on ball attachments on the restant teeth and overdenture on mini implants at the mandibula (Mini Sky1 Bredent) in order to offer the dynamic balance of the oral rehabilitation. In order to apply the mini implants in the optimal position as anatomical rapports but also from the prosthodontic point of view related to the occlusal relationships, the antagonist dental arch and to obtain the mandibular balance conditions, we used the full conventional denture to obtain an implanto-prosthodontic guide. 

 The surgical guide, implant - prosthodontic was done in the denture’s base. After we obtained the mandibular full denture, this was used to make a teleradiography with an opaque substance. X-resin Flow (Bredent) radio opaque substance was used. The used substance is low consistency silicon with radio opaque properties; with it, important elements are highlighted in the X-rays. The product is packed in two auto-mixing tubes for a precise dosage. The mixing is obtained by pushing the content of the 2 tubes trough a mixing canola using a piston. To carry off the silicon from the denture is very simple using alcohol [**4**].

**Fig. 8:**
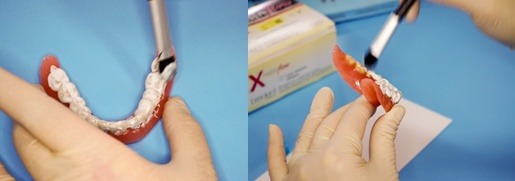
Apllying the X-resin on the denture’s teeth

**Fig. 9:**
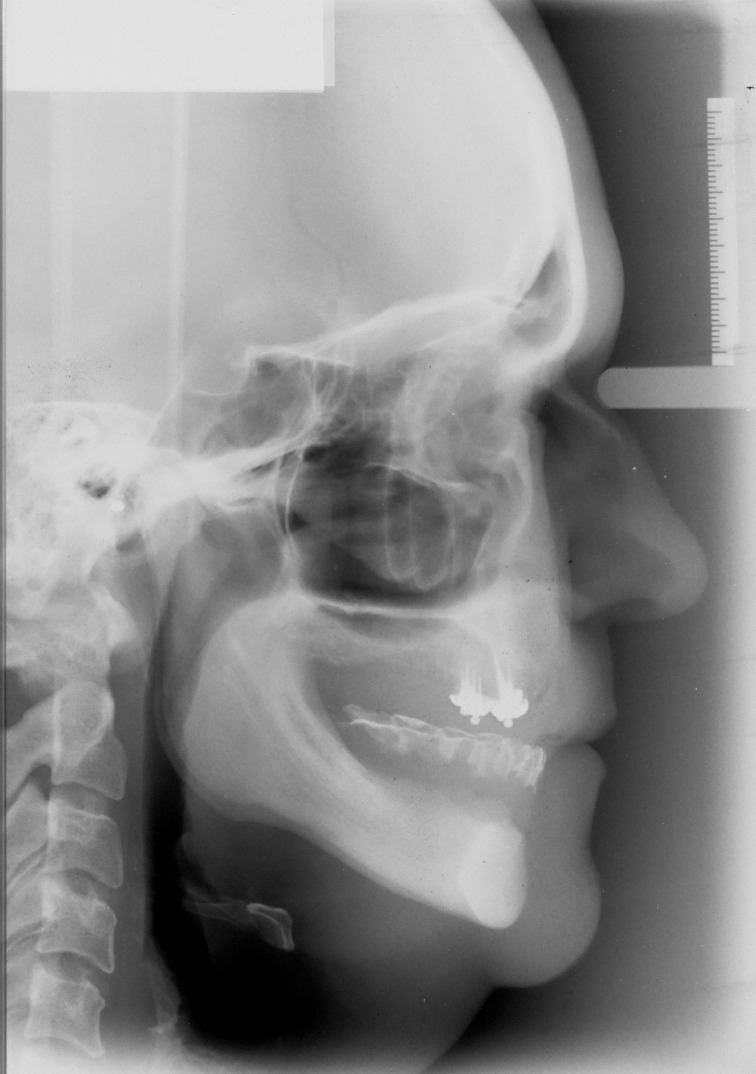
Profile teleradiography with the radiopacised denture

 a) To verify the denture, on the artificial dental arches we pensulated X-resin flow in a thin film and then we waited for 5 minutes to indurate (**Fig. 8**). The profile teleradiography was done with the mandibular denture covered with X-resin flow, the denture being on the prosthodontic field, in order to verify the teeth montage, in harmony with the cranio-facial markers and soft facial parts (**Fig. 9**).

**Fig. 10:**
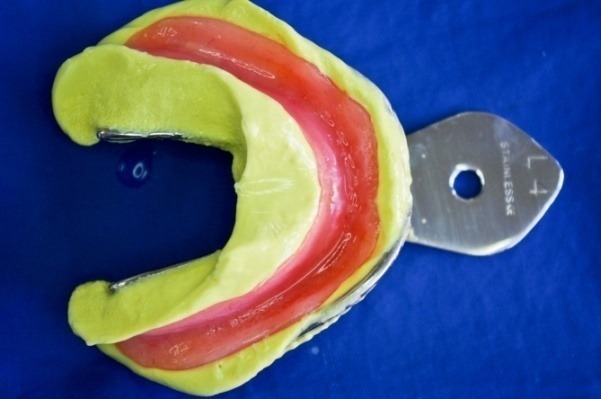
The denture’s impression

**Fig. 11:**
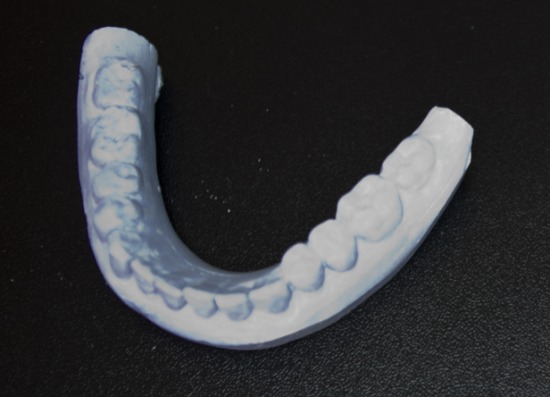
The plaster cast

**Fig. 12:**
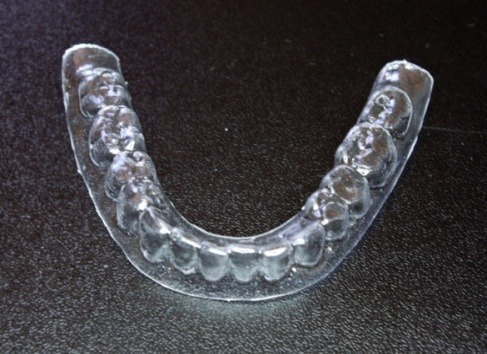
The surgical guide

 Obtaining the surgical guide. In a first phase, we have obtained the impression of the mandibular denture with irreversible hydrocolloid- alginate (**Fig. 10**). The denture was in the patient’s mouth. After the casting (**Fig. 11**), we made a transparent occlusal guard that was used as a surgical guide to position optimally the mini-implants, using the whole information obtained using the ortopantomography and teleradiography (**Fig. 12**).

 The clinical phases of the mini implant application using the radio opaque guide

**Fig. 13:**
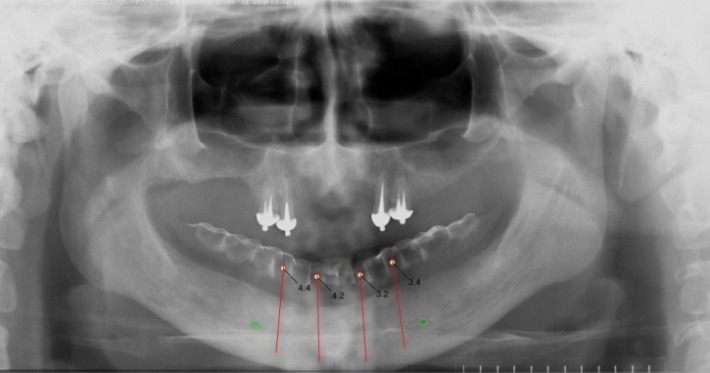
The placement of the mini implants using the X-ray guide

**Fig. 14:**
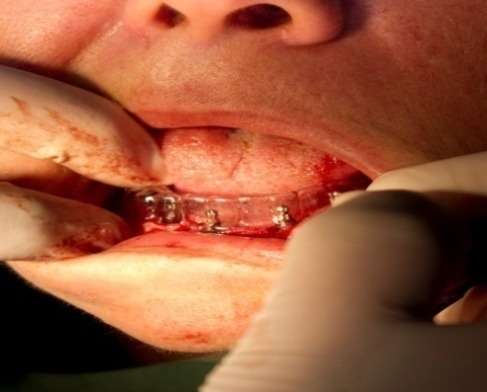
The placement of the mini implants

 The placement of the mandibular mini implants was planned after the ortopantomography (**Fig. 13**) with the mandibular denture covered with the radio opacque paste, in the mouth. The placement of 4 interforaminal mini implants was decided, in a correct relation with the denture’s teeth (**Fig. 14**).

 Having in mind the oseous offer, mini implants of 2.8 mm in diameter and lenght of 14 mm were chosen. To place the mini implants the surgical guide, obtaines with the imaging ortopantomographyc method and teleradiographyc was used. 

**Fig. 15:**
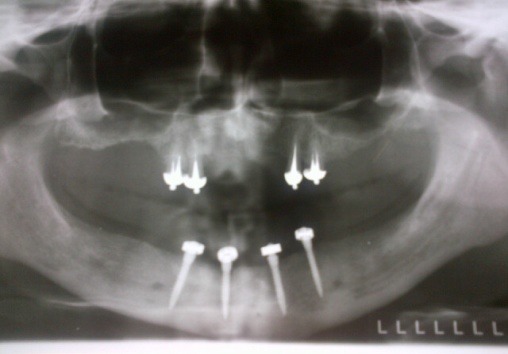
Rx control after the osseous integration

**Fig. 16:**
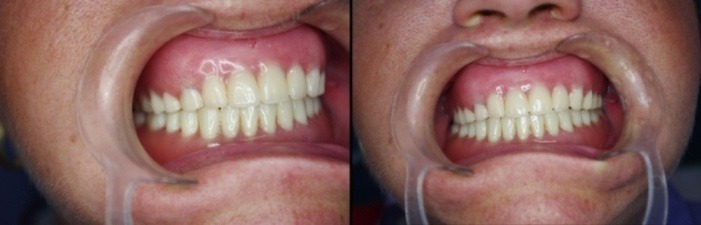
Intraoral control of the loaded dentures (with the applied o-rings)

 After the osseous integration of the mini implants, the overdenture’s treatment was done following the protocol, trespectiv the pacient wore for 3 months the conventional dentures, and then the mini implants were loaded by applying the o – rings in the denture’s base. The result was a well integrated implanto- prosthodontic rehabilitation, functionally and esthetically [**6,7**] with the osseous parts, the facial esthetics and antagonic arch, as prooved by the clinical intra- and extra- oral, and the Rx ortopantomographyc control (**Fig. 15,Fig. 16**).

## Results and discussions

The method’s importance is obvious especially in the complex clinical cases, borderline cases, where the diagnosis is specified, simplifies and confirms the clinical diagnosis.

 The method offers information regarding the relationship between the cover soft parts, of the facial skeleton. We have to keep in mind the fact that the soft parts suffer an important fluctuance regarding the thickness at some levels and the profile may be different influenced. The collected data regarding the thickness of the soft part will have a role in establishing the diagnosis and prognostication, sometimes in conducting the treatment.

These imaging exams may help to establish the treatment, offering information regarding the most adequate treatment type and its efficiency in time. 

 The teleradiography may offer a valuable esthetic prognostication, as Tigelkamp noticed “the profile changes don’t correspond always to the operator’s illusions, to the imagined picture speaking about the maxillary changes, although, generally the soft parts are almost like the osseous profile and their changes are similar to the skeletal ones" [**3**].

 The profile teleradiographie’s references (instructions), in full edentulism cases, may be general (diagnosis) or local, especially for a step of the treatment. 

 As general indications, we remind: 

• The establishment with certitude of the intermaxilary relationships, mandibular prognathism/ retrognathism;

• The skeletal asymmetries highlight;

 • Appreciations about the soft part’s contour.

 As local indications, we remind:

 • The periodic control of the clinical value of the complete dentures [**1**];

 • The orientation of the prosthodontic occlusal plane in the office and laboratory;

 • The dimension of the lower segment of the face, reported to the figure and crane.

 • Evaluation of the correct position of the anterior artificial teeth (incisors) and physiologic occlusal space

 • The radiologic confirmation of a correct relation mandibulo-cranial, with a study of the condylian trajectory and mandibular positions

 The lateral teleradiography with the complete dentures on occlusion which artificial teeth are visible by the application of the radio opaque silicon paste allows studying the following cephalometric parameters:

 1. The face’s depht related to the facial angle;

 2. The anterior face’s height related to the facial axis;

 3. The facial convexity analised by the reference of the point A to the N-Pg plane; 

 4. The position of the front teeth related to the A-Pg ;

 5. The inferior incisors’s inclination is measures reported to A-Pg line

 6. The position of the lips towards Ricketts esthetic line;

 7. The garanty of the right positionning of artificial teeth, determined by objective criterias as evidenced in littérature [8,9], respecting the relation between artificial teeth, anatomical structures associated with the right placement of mini implants represent a challenge for the doctors. This is the main advantage of this original method allowing the visualisatin on the same image of the 3 elements involved: artificial teeth, anatomical structures and implants position.

## Conclusions

The usefull interest oh the teleradiography with radio opac substance is certainly evident on conventionnal complete denture and especially on implants overdentures, these 2 radiological investigations may eeven some times replace the more invasive and cost full computer tomograph technique. These methods provides :

 1. Visualisation of the right teeth positionning and even the relation between the overdenture base and the support structures.

 2. The optimal positionning of implants/mini implants related to anatomical structures and prosthetic requirements.

 3. The use of the conventionnal denture for the realisation of the surgical guide allows the correct implants placement prosthetically and anatomical driven.

 4. This is an easy method, accessible to any patient and practicionner, with minimal costs, providing important benefits to the successfull therapy of the complete edentulism.

 5. These imaging techniques confer to the therapy of full edentulism the evidence-based arguments recquired by modern dentistry standards.

 6. Easy, accessible and minimal costs techniques with large applicability on the implant prosthetic procedures.
